# Hypoxanthine in the microenvironment can enable thiopurine resistance in acute lymphoblastic leukemia

**DOI:** 10.3389/fonc.2024.1440650

**Published:** 2024-07-19

**Authors:** Xiaohong Wang, Jason Ostergaard, Jongseok Kang, Grace Sagong, Rachel Twite, Andrea Vargas-Morales, Peter M. Gordon

**Affiliations:** ^1^ Division of Pediatric Hematology and Oncology, Department of Pediatrics, University of Minnesota, Minneapolis, MN, United States; ^2^ Masonic Cancer Center, University of Minnesota, Minneapolis, MN, United States

**Keywords:** acute lymphoblastic leukemia, chemoresistance, thiopurine compounds, microenvironment, tissue culture media

## Abstract

Acute lymphoblastic leukemia (ALL) is the most common pediatric malignancy, with relapse being a major obstacle to successful treatment. Our understanding of the mechanisms driving chemotherapy resistance and ultimately relapse in leukemia remains incomplete. Herein, we investigate the impact of the tumor microenvironment on leukemia cell drug responses using human plasma-like media (HPLM), designed to mimic physiological conditions more accurately *ex vivo*. We demonstrate that while most chemotherapeutics maintain an efficacy in HPLM comparable to standard tissue culture media, the thiopurines 6-mercaptopurine (6-MP) and 6-thioguanine (6-TG) exhibit significantly reduced potency and efficacy against both B- and T- leukemia cells in HPLM. By merging our understanding of thiopurines’ mechanism of action with the metabolites supplemented in HPLM compared to standard media, we proposed and subsequently validated the hypothesis that hypoxanthine, a purine derivative, is responsible for conferring resistance to the thiopurines. Importantly, the concentration of hypoxanthine required for resistance is comparable to physiological levels found *in vivo*, supporting clinical relevance. Our findings demonstrate the utility of a more physiologic media in identifying and characterizing mechanisms by which the microenvironment can enable resistance. Understanding such interactions may inform strategies to overcome drug resistance and improve therapeutic outcomes in pediatric leukemia.

## Introduction

Acute lymphoblastic leukemia (ALL) is the most common childhood malignancy. Despite significant advances in leukemia therapy, approximately 15-20% of pediatric patients experience disease relapse (1). As a result, relapse is the most common cause of treatment failure in ALL and relapsed ALL is as common as most pediatric solid tumors and acute myeloid leukemia in childhood. Relapsed leukemia is often refractory to conventional chemotherapy and confers a poor prognosis. How leukemia cells escape the effects of multi-agent chemotherapy and persist during several years of treatment is incompletely understood but likely a combination of genetic, epigenetic, and metabolic mechanisms of drug resistance (2–5).

Accordingly, identifying mechanisms of leukemia chemoresistance is critical for developing more efficacious therapies that fully eradicate the disease*. Ex vivo* drug testing in tissue culture is a rapid and economical approach for elucidating drug responses and mechanisms of action that can provide a crucial foundation for subsequent *in vivo* investigations and clinical trials. Nonetheless, a drawback of this approach lies in traditional tissue culture media, originally designed to support cancer cell growth *ex vivo* rather than replicate the intricate tumor microenvironment *in vivo*. Consequently, conventional tissue culture approaches may fail to identify environmentally driven metabolic adaptations in cancer cells that can enable drug resistance. However, recent developments of commercially available and affordable tissue culture media supplemented with metabolites found in human plasma (human plasma-like media) have facilitated the discovery of novel and physiologically relevant mechanisms of drug resistance previously obscured by traditional media formulations (6–11).

Herein, we compared the efficacy of multiple chemotherapeutics used in ALL therapy in regular tissue culture media with human plasma-like media (HPLM). Using this approach, we identified the purine metabolite hypoxanthine as a leukemia cell extrinsic mediator of thiopurine resistance.

## Methods

### Cell culture

Leukemia cell lines were obtained from American Type Culture Collection (ATCC) or DSMZ. Leukemia cell lines were cultured in RPMI or human plasma-like media (ThermoFisher Scientific) supplemented with regular or dialyzed Fetal Bovine Serum (FBS) 10% and Penicillin-Streptomycin. Every two months leukemia cells in culture were replaced with new cells from the original expansion.

### Drugs and reagents

6-mercaptopurine, 6-thioguanine, cytarabine, doxorubicin, L-asparaginase, vincristine, etoposide, and clofarabine were purchased from MedChemExpress. Hypoxanthine was from Sigma-Aldrich. Betaine was from Selleck Chemicals.

### Proliferation and apoptosis assays

Leukemia cell proliferation and viability were assessed with the CellTiter-Glo Luminescent Cell Viability Assay (Promega) and a Tecan Infinite M200 Pro plate reader. All experiments were performed with at least 3 wells per condition. Leukemia cell apoptosis was measured using the Caspase-Glo 3/7Assay (Promega), according to the manufacturer’s instructions.

### Statistical analysis

Results are shown as the mean plus or minus the SD. All experiments were performed at least twice, and most often at least three times, with representative data of one experiment presented. Student’s t-test or ANOVA were used for statistical comparisons between groups and are described in the figure legends. *P*-values < 0.05 were considered statistically significant. All graphing, curve fitting [non-linear regression, sigmoidal, four-parameter logistic, X is log(concentration)], and statistical significance testing were performed using GraphPad Prism 10.1.1 software (GraphPad Software, La Jolla, CA).

## Results

**Figure 1:**
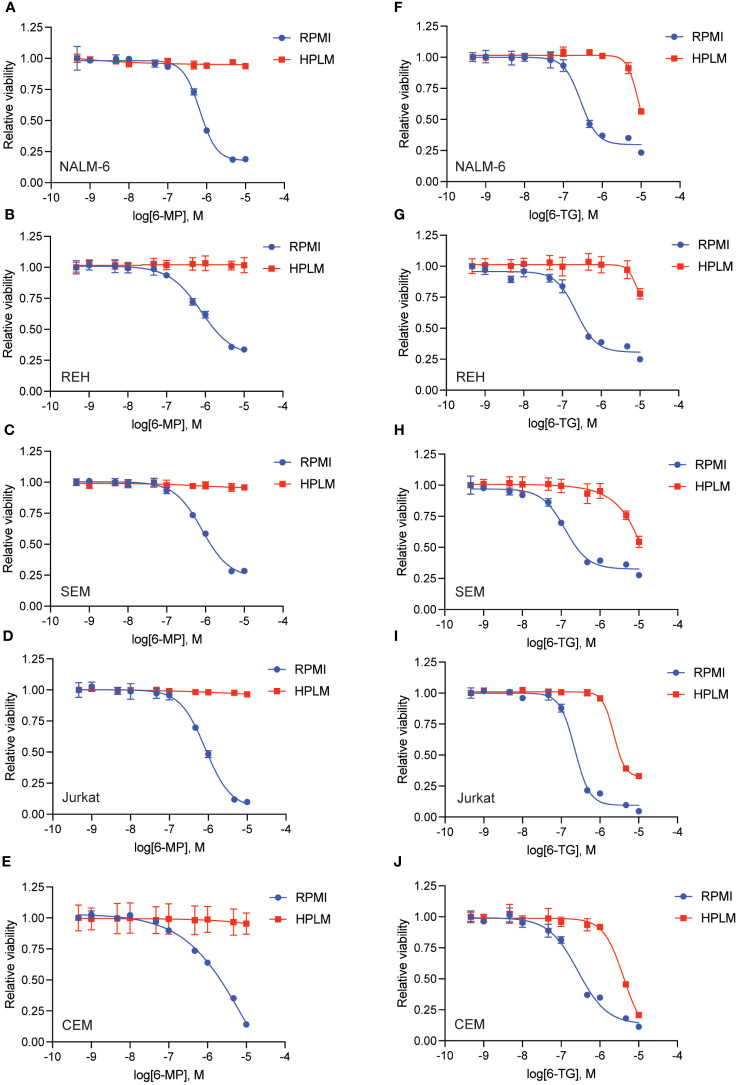
Human plasma-like media (HPLM) attenuates leukemia cell sensitivity to thiopurine chemotherapeutics. 6-MP **(A–E)** and 6-TG **(F–J)** dose-response curves for leukemia cells (NALM-6, **A**, **F**; REH, **B**, **G**; SEM, **C**, **H**; Jurkat, **D**, **I**; CEM, **E**, **J**) in regular or HPLM media. Leukemia cell viability was assessed after 48 hours of drug treatment using the CellTiter-Glo Luminescent Cell Viability Assay. Error bars represent the mean ± SD of three technical replicates.

We generated dose-response curves for multiple chemotherapeutics used in ALL therapy with both B- (NALM-6, REH, SEM) and T-ALL (CEM, Jurkat) cell lines in either regular (RPMI) or human plasma-like media (HPLM). Drugs tested included doxorubicin, 6-mercaptopurine (6-MP), vincristine, cytarabine, asparaginase, etoposide, and clofarabine. While all the leukemia cells line were very sensitive to 6-MP in regular media they became almost completely resistant in HPLM (**Figures 1A–E**). For the other drugs tested there was very little difference in leukemia cell sensitivity in regular media compared to HPLM, except for cytarabine and asparaginase for which several, but not all, cell lines were moderately less sensitive to both drugs in HPLM media (**Supplementary Figures 1**-**3**).

**Figure 2:**
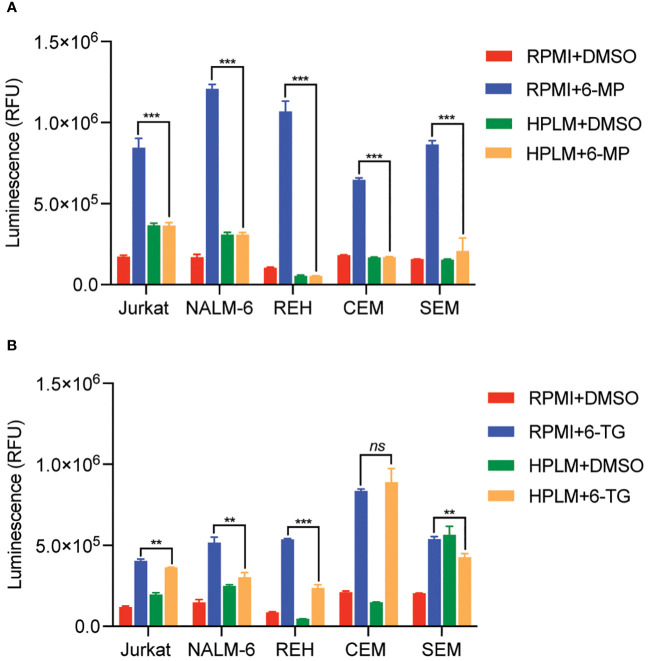
Human plasma-like media (HPLM) attenuates thiopurine-mediated leukemia cell apoptosis. Caspase 3/7 activity was assessed in leukemia cells cultured in HPLM or regular media after 48 hours of treatment with either 6-MP 10 μM **(A)** or 6-TG 10 μM **(B)**. Error bars represent the mean ± SD of three technical replicates. *P*: **, <0.01 and ***, <0.001 by ANOVA. ns, not significant.

We next tested whether this HPLM-mediated chemoresistance extended to other thiopurine antimetabolites drugs in addition to 6-MP. Accordingly, we generated 6-thioguanine (6-TG) dose-response curves in both regular and HPLM media. Leukemia cells were significantly more resistant to 6-TG in HPLM compared to regular media, supporting a generalizable effect of HPLM on thiopurine antimetabolite resistance (**Figures 1F–J**). However, the effect of HPLM on 6-TG was slightly more modest than that seen with 6-MP. We next measured caspase activity in leukemia cells to further assess whether HPLM was rescuing the effects of thiopurines on apoptosis specifically. In agreement with the prior data, 6-MP and 6-TG caused significantly less caspase 3/7 activation in leukemia cells in HPLM relative to regular media. (**Figures 2A, B**).

**Figure 3:**
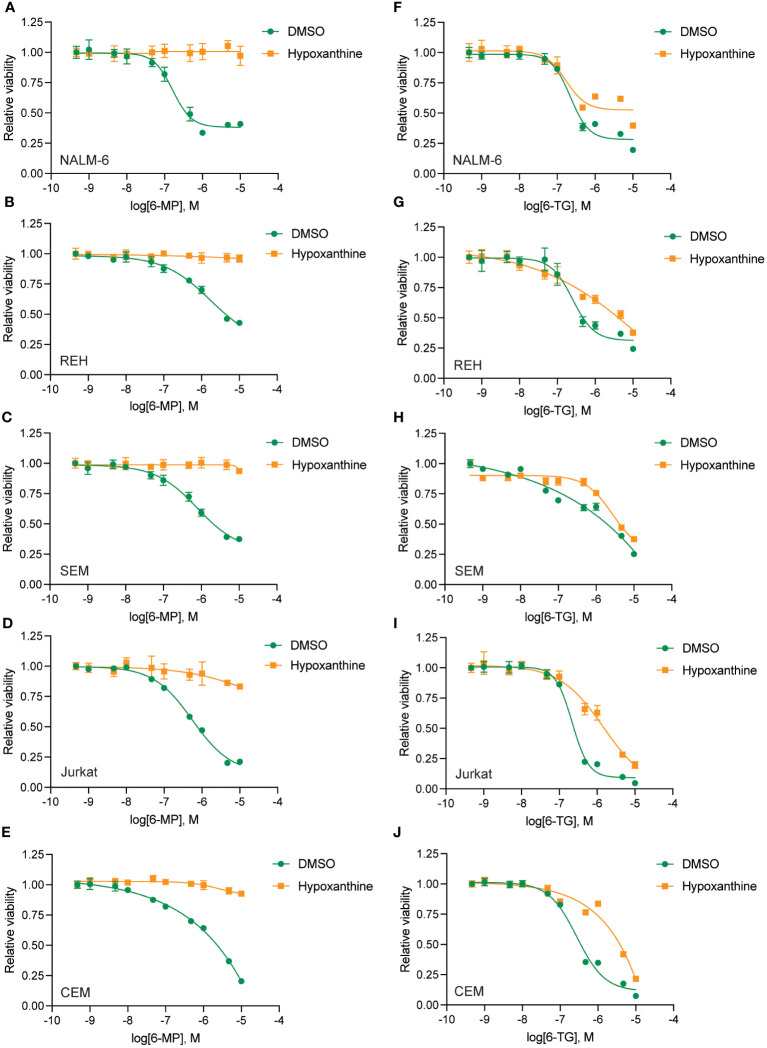
Hypoxanthine supplementation of regular media enhances leukemia cell resistance to thiopurines. 6-MP **(A–E)** and 6-TG **(F–J)** dose-response curves for leukemia cells (NALM-6, **A**, **F**; REH, **B**, **G**; SEM, **C**, **H**; Jurkat, **D**, **I**; CEM, **E**, **J**) in regular media supplemented with hypoxanthine or DMSO control. Leukemia cell viability was assessed after 48 hours of drug treatment using the CellTiter-Glo Luminescent Cell Viability Assay. Error bars represent the mean ± SD of three technical replicates.

To define the mechanism(s) of HPLM-mediated thiopurine resistance we compared the components of RPMI and HPLM media. We identified hypoxanthine and betaine as compounds unique to HPLM media that potentially could modulate the mechanism of action for thiopurines (12–15). Hypoxanthine is a purine derivative and an intermediate in adenosine metabolism and in the formation of purine nucleic acids by the salvage pathway. Similarly, betaine is a modified amino acid that serves as a methyl donor for the regeneration of S-adenosyl methionine (SAM) and the subsequent metabolic detoxification of thiopurines. We next generated 6-MP and 6-TG dose-response curves in regular media supplemented with either betaine or hypoxanthine to test whether either of these metabolites could enable thiopurine resistance. Betaine had no effect on the sensitivity of leukemia cells to either 6-MP or 6-TG (**Supplementary Figure 4**). In contrast, hypoxanthine caused significant resistance to 6-MP (**Figures 3A–E**) and, to a lesser extent, 6-TG (**Figures 3F–J**).

**Figure 4:**
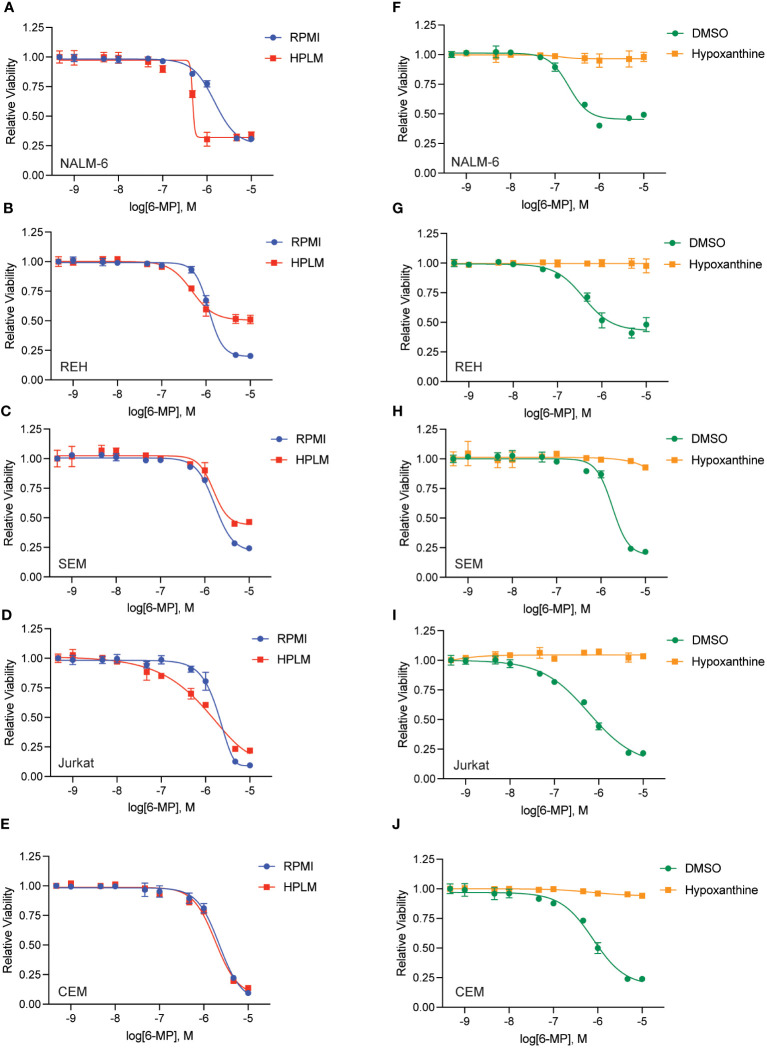
Hypoxanthine in HPLM media contributes to 6-MP resistance. **(A–E)** 6-MP dose-response curves for leukemia cells (NALM-6, **A**; REH, **B**; SEM, **C**; Jurkat, **D**; CEM, **E**) pre-incubated in HPLM or RPMI for 48 hours prior to the addition of 6-MP. After an additional 48 hours of drug treatment, leukemia cell viability was assessed using the CellTiter-Glo Luminescent Cell Viability Assay. Error bars represent the mean ± SD of three technical replicates. **(F–J)** 6-MP dose-response curves for leukemia cells (NALM-6, **F**; REH, **G**; SEM, **H**; Jurkat, **I**; CEM, **J**) pre-incubated in HPLM for 48 hours prior to the addition of 6-MP +/- hypoxanthine 10 μM. After an additional 48 hours of drug treatment, leukemia cell viability was assessed using the CellTiter-Glo Luminescent Cell Viability Assay. Error bars represent the mean ± SD of three technical replicates.

**Figure 5:**
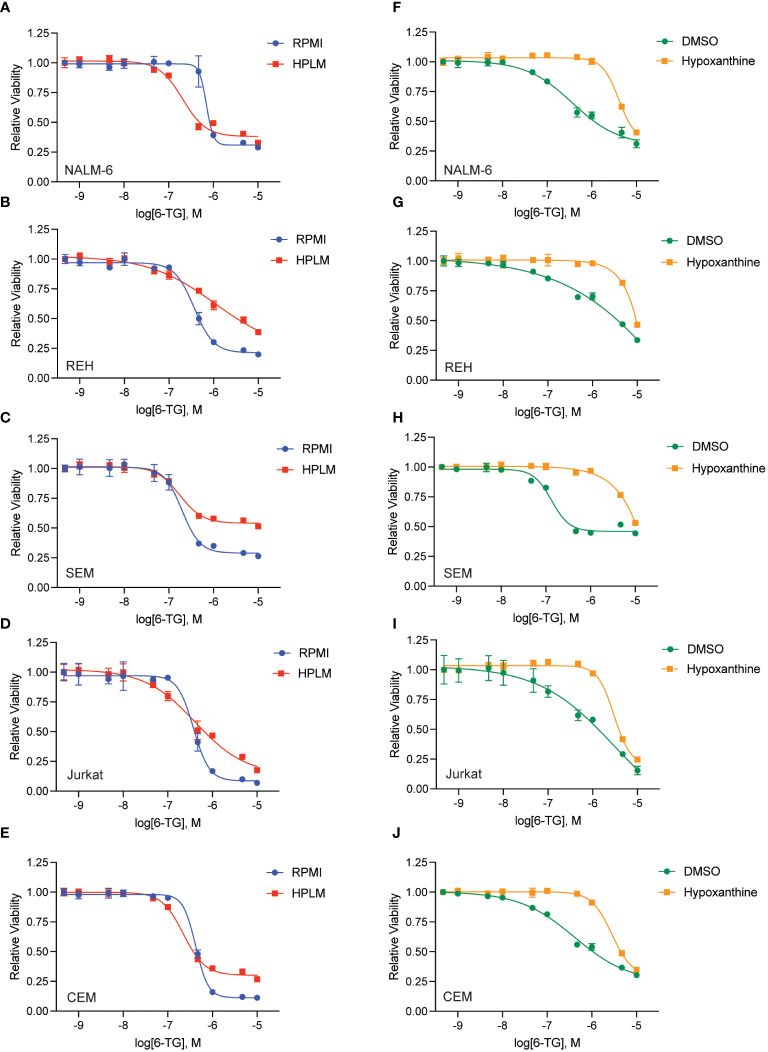
Hypoxanthine in HPLM media contributes to 6-TG resistance. **(A–E)** 6-TG dose-response curves for leukemia cells (NALM-6, **A**; REH, **B**; SEM, **C**; Jurkat, **D**; CEM, **E**) pre-incubated in HPLM or RPMI for 48 hours prior to the addition of 6-TG. After an additional 48 hours of drug treatment, leukemia cell viability was assessed using the CellTiter-Glo Luminescent Cell Viability Assay. Error bars represent the mean ± SD of three technical replicates. **(F–J)** 6-TG dose-response curves for leukemia cells (NALM-6, **F**; REH, **G**; SEM, **H**; Jurkat, **I**; CEM, **J**) pre-incubated in HPLM for 48 hours prior to the addition of 6-TG +/- hypoxanthine 10 μM. After an additional 48 hours of drug treatment, leukemia cell viability was assessed using the CellTiter-Glo Luminescent Cell Viability Assay. Error bars represent the mean ± SD of three technical replicates.

**Figure 6:**
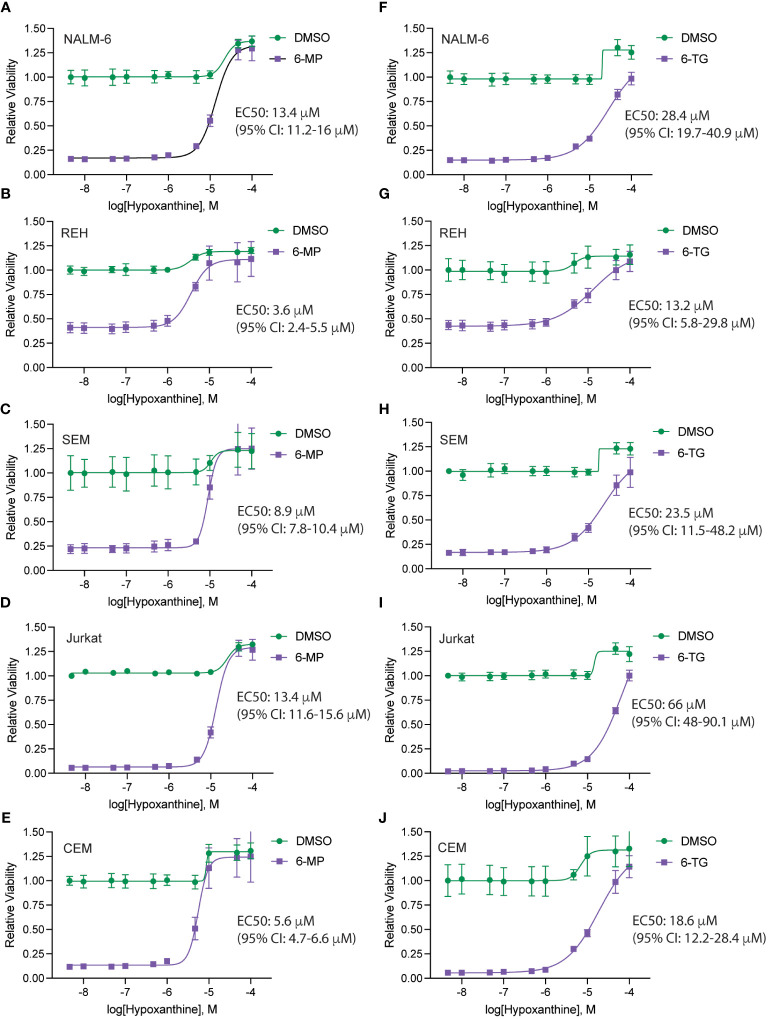
Hypoxanthine concentrations required to rescue thiopurine toxicity. Hypoxanthine dose-response curves for leukemia cells (NALM-6, **A**, **F**; REH, **B**, **G**; SEM, **C**, **H**; Jurkat, **D**, **I**; CEM, **E**, **J**) in the presence of 6-MP 4.7 μM **(A–E)** or 6-TG 4.7 μM **(F–J)**. Leukemia cell viability was assessed after 48 hours of drug treatment using the CellTiter-Glo Luminescent Cell Viability Assay. Error bars represent the mean ± SD of three technical replicates. EC50 values with confidence intervals were calculated from the dose-response curves and are shown for each cell line.

To further test whether hypoxanthine within HPLM media is enabling thiopurine resistance we capitalized on the prior observation that highly proliferative cells rapidly utilize and deplete hypoxanthine in tissue culture (16). Accordingly, we cultured leukemia cells in media in the absence of any drugs for 48 hours to allow for hypoxanthine depletion (‘pre-incubation’) prior to adding thiopurines in the presence or absence of hypoxanthine for an additional 48 hours (**Figures 4**, **5**). Pre-incubation in HPLM media restored leukemia cell sensitivity to 6-MP (**Figures 4A–E**) and 6-TG (**Figures 5A–E**) to an extent that was relatively comparable to that seen in regular media. However, if hypoxanthine was supplemented after the pre-incubation period, the effect of pre-incubation was reversed, and the leukemia cells were again significantly resistant to both 6-MP (**Figures 4F–J**) and 6-TG (**Figures 5F–J**). Together, these experiments support that hypoxanthine within HPLM can rescue thiopurine toxicity in leukemia cells.

Finally, to further assess if these results are potentially clinically applicable, we asked whether the hypoxanthine concentration required for rescuing thiopurine toxicity was comparable to hypoxanthine levels measured *in vivo*. Accordingly, we generated hypoxanthine dose-response curves in the absence and presence of a fixed thiopurine dose that caused ~80-90% cell death (**Figure 6**). For these experiments we also used dialyzed FBS as dialysis removes any hypoxanthine present in the FBS. In agreement with our prior data, hypoxanthine completely rescued the effects of the thiopurine toxicity at higher doses. The hypoxanthine EC50 values calculated from the dose-response curves were in the ~5-15 μM range for 6-MP and moderately higher for 6-TG. For 6-MP, in particular, this is a physiologically relevant hypoxanthine concentration and support that *in vivo* hypoxanthine may modulate thiopurine efficacy in eradicating leukemia cells (17–19).

## Discussion

Soluble factors in the tumor microenvironment, such as proteins and metabolites, can exert a critical influence on cancer cell drug resistance (2, 20). Soluble factors achieve this by diverse mechanisms including altering drug uptake and metabolic pathways, as well as by regulating cancer cell signaling pathways that drive drug resistance. Unlike genetic or epigenetic mechanisms of drug resistance, the effects of these soluble factors on cancer cells are often reversible. As a result, removal or effective targeting of these factors can often restore cancer cell drug sensitivity.

To identify soluble factors impacting drug resistance in ALL, we assessed the effectiveness of multiple chemotherapeutic agents commonly used in ALL therapy in a commercially available human plasma-like medium (HPLM). This medium was designed to mimic the metabolite composition of human plasma more closely than traditional tissue culture media (6–10). While the addition of these metabolites had minimal impact on most of the drugs tested, we observed a significant attenuation of the potency and efficacy of 6-mercaptopurine (6-MP) and 6-thioguanine (6-TG) in HPLM. 6-MP has been a cornerstone of ALL therapy for over 60 years and is administered daily during the critical maintenance phase of therapy necessary for long-term cures (21). Notably, poor adherence to chemotherapy (< 90%–95%) during maintenance therapy has been linked to a substantial increase in relapse risk, underscoring the importance of 6-MP in achieving cure (21, 22).

6-MP and 6-TG are thiopurines that target the purine pathway and DNA synthesis. Both drugs undergo several metabolic conversions prior to incorporation into DNA which then triggers induction of the DNA damage response and apoptosis (21). By comparing the metabolites supplemented in HPLM relative to regular media in the context of thiopurines’ mechanism of action we quickly hypothesized, and subsequently confirmed, that hypoxanthine within HPLM mediated thiopurine resistance. Hypoxanthine, a purine derivative, is produced in the metabolism of adenosine but can also be utilized in the purine salvage pathway that complements *de novo* purine synthesis (15). Whole exome sequencing in relapsed ALL patients recently identified mutations in phosphoribosyl pyrophosphate synthetase 1 (*PRPS1*), a key enzyme in the purine synthesis pathway (23). *PRPS1* mutations conferred thiopurine resistance through activation of *de novo* purine synthesis and accumulation of intracellular hypoxanthine that competitively inhibited 6-MP conversion and subsequent DNA damage. Of note, 6-MP undergoes several additional enzymatic activation steps than 6-TG which may contribute to the increased hypoxanthine-mediated resistance to 6-MP than 6-TG that we observed in our cytotoxicity assays (21, 24).

Our findings complement this genetic work and suggest that hypoxanthine in the leukemia microenvironment can also attenuate thiopurine efficacy. In agreement, it was previously shown that hypoxanthine had similar effects on a single acute promyelocytic leukemia cell line (HL-60) (25). Importantly, we demonstrated that 6-MP resistance induced by hypoxanthine occurs at concentrations similar to those found in plasma. Additionally, hypoxanthine levels vary significantly across tissues and under various physiological and pathological conditions, including several that have been shown to impact ALL biology and treatment outcomes. In the bone marrow, hypoxanthine levels are up to 10-fold higher than in venous blood, while in cerebral spinal fluid hypoxanthine levels are elevated by 2-8-fold compared to venous blood (17). Notably, both the bone marrow and the central nervous system are frequent sites of leukemia relapse as well as relatively hypoxic environments, which has been associated with hypoxanthine elevations.

Similarly, obesity and adipose tissue have also been associated with elevated hypoxanthine levels (26–28), suggesting that hypoxanthine may also contribute to other well described mechanisms by which obesity enables leukemia chemoresistance (29). In pediatric/AYA ALL patients, obesity at diagnosis is associated with higher minimal residual disease (MRD) at the end of induction and lower disease-free survival (30, 31). Body mass index (BMI) also tends to rise significantly during ALL treatment (32, 33). Extreme obesity during maintenance therapy, when 6-MP is taken daily, was associated with a greater relapse risk and lower levels of erythrocyte thioguanine nucleotides, even after adjusting for adherence to oral chemotherapy and other potential confounding factors (34). However, it is worth noting that in this study these lower erythrocyte thioguanine levels did not explain the greater hazard of relapse. Although the mechanisms underlying ALL therapy resistance in the bone marrow, central nervous system, and obesity are complex and involve multiple factors, the evidence provided here indicates that hypoxanthine within various leukemia microenvironments could potentially influence both the effectiveness and resistance to thiopurine treatment in ALL therapy.

Accordingly, addressing these physiological processes that increase hypoxanthine, such as obesity or hypoxia secondary to obstructive sleep apnea (26, 27, 35), could potentially increase the efficacy of thiopurines in ALL therapy. Pharmacological or enzymatic approaches could also be used to target hypoxanthine levels, but this may be challenging due to the complexity of the purine synthesis, salvage and metabolic pathways. For example, while xanthine oxidase inhibitors, such as allopurinol, increase hypoxanthine levels, there is an abundance of clinical data demonstrating that allopurinol increases, rather than decreases, the potency of 6-MP by skewing its metabolism (36). Finally, this work also demonstrates the utility of physiologic media in identifying and characterizing mechanisms by which the microenvironment can enable drug resistance.

## Data availability statement

The original contributions presented in the study are included in the article/**Supplementary Material**. Further inquiries can be directed to the corresponding author.

## Ethics statement

Ethical approval was not required for the studies on humans in accordance with the local legislation and institutional requirements because only commercially available established cell lines were used. Ethical approval was not required for the studies on animals in accordance with the local legislation and institutional requirements because only commercially available established cell lines were used.

## Author contributions

XW: Data curation, Formal analysis, Investigation, Methodology, Validation, Visualization, Writing – review & editing. JO: Data curation, Formal analysis, Investigation, Methodology, Visualization, Writing – review & editing. JK: Data curation, Formal analysis, Investigation, Methodology, Visualization, Writing – review & editing. GS: Data curation, Investigation, Writing – review & editing. RT: Data curation, Investigation, Writing – review & editing. AV-M: Data curation, Investigation, Writing – review & editing. PG: Conceptualization, Data curation, Formal analysis, Funding acquisition, Investigation, Methodology, Project administration, Resources, Supervision, Writing – original draft, Writing – review & editing.
