# Supplementary material for: Hypoxanthine in the microenvironment can enable thiopurine resistance in acute lymphoblastic leukemia
Source: Front Oncol. 2024 Jul 19;14:1440650. doi: 10.3389/fonc.2024.1440650 (PMC11294174; doi:10.3389/fonc.2024.1440650)
Supplement: Supplementary file 1 [file Image_1.pdf]

Supplemental Figure 1

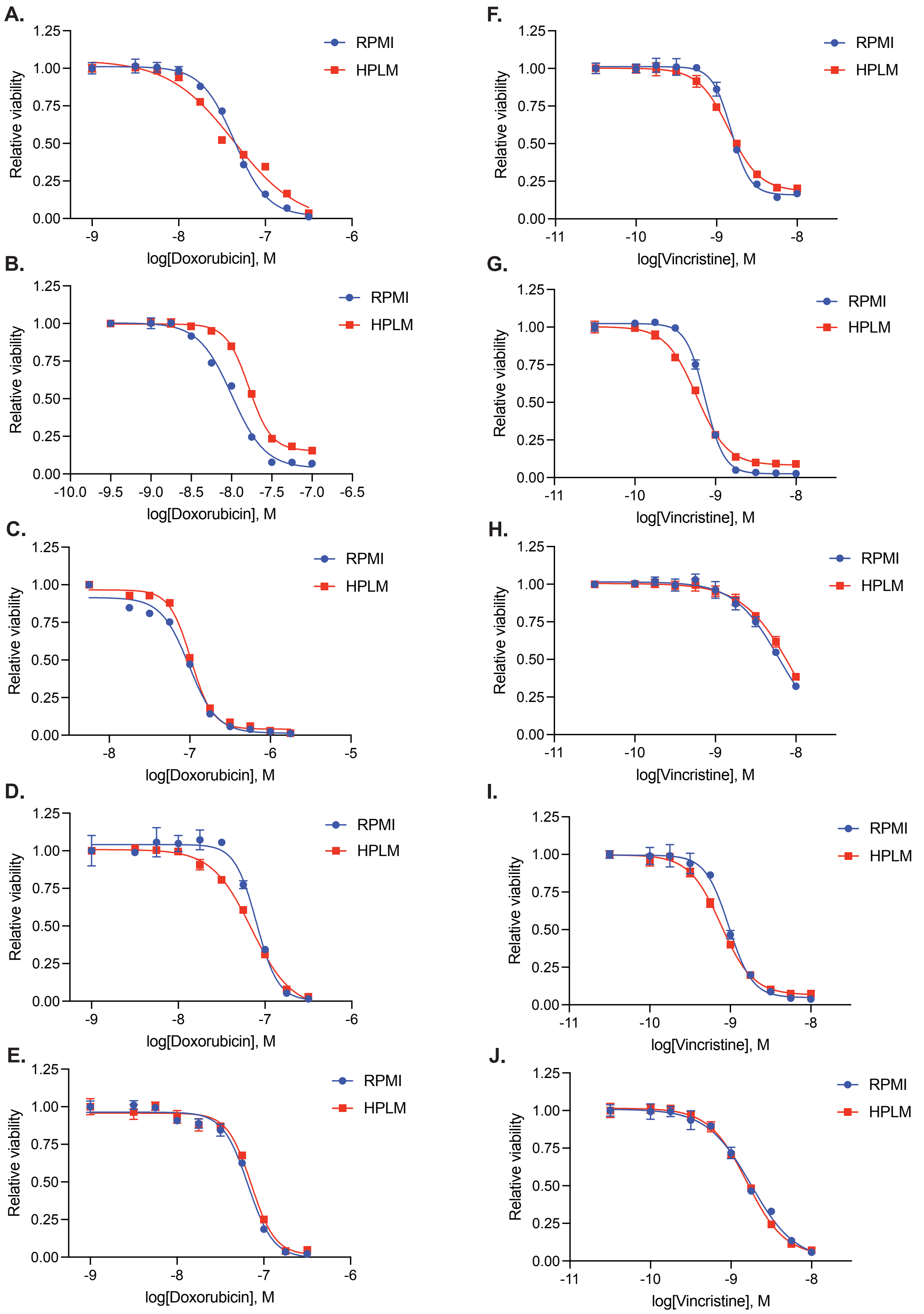

Supplemental Figure 2

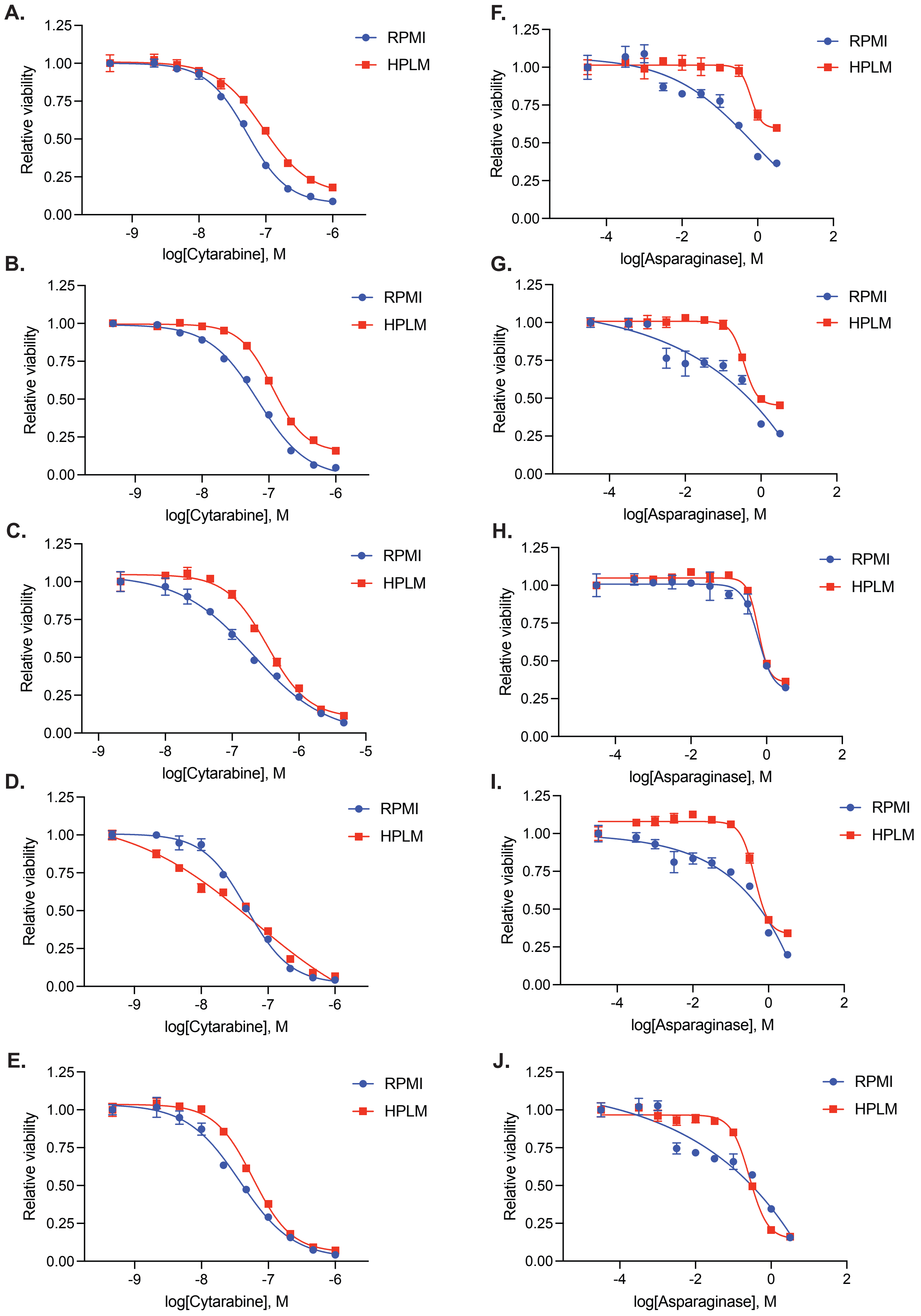

Supplemental Figure 3

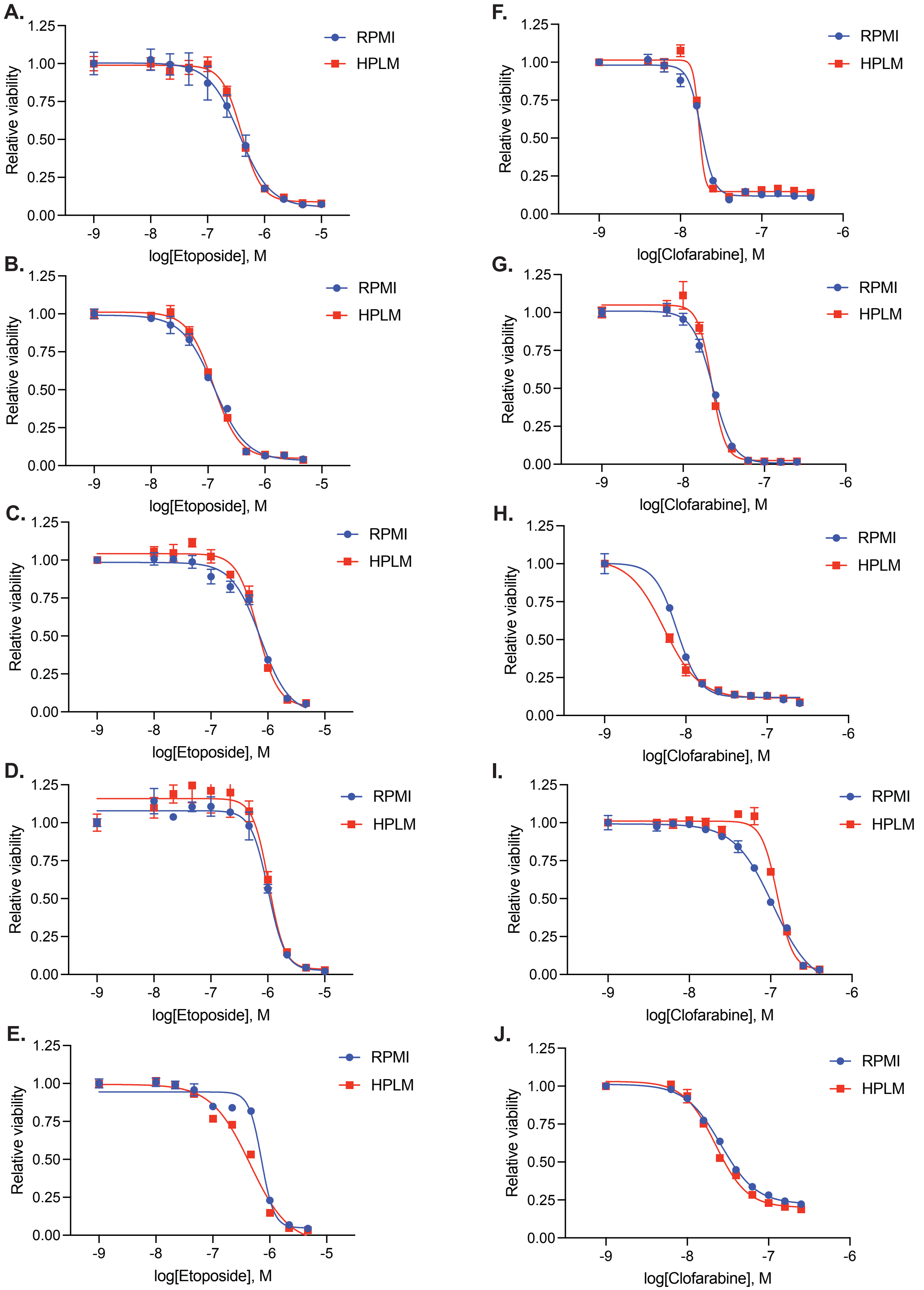

Supplemental Figure 4

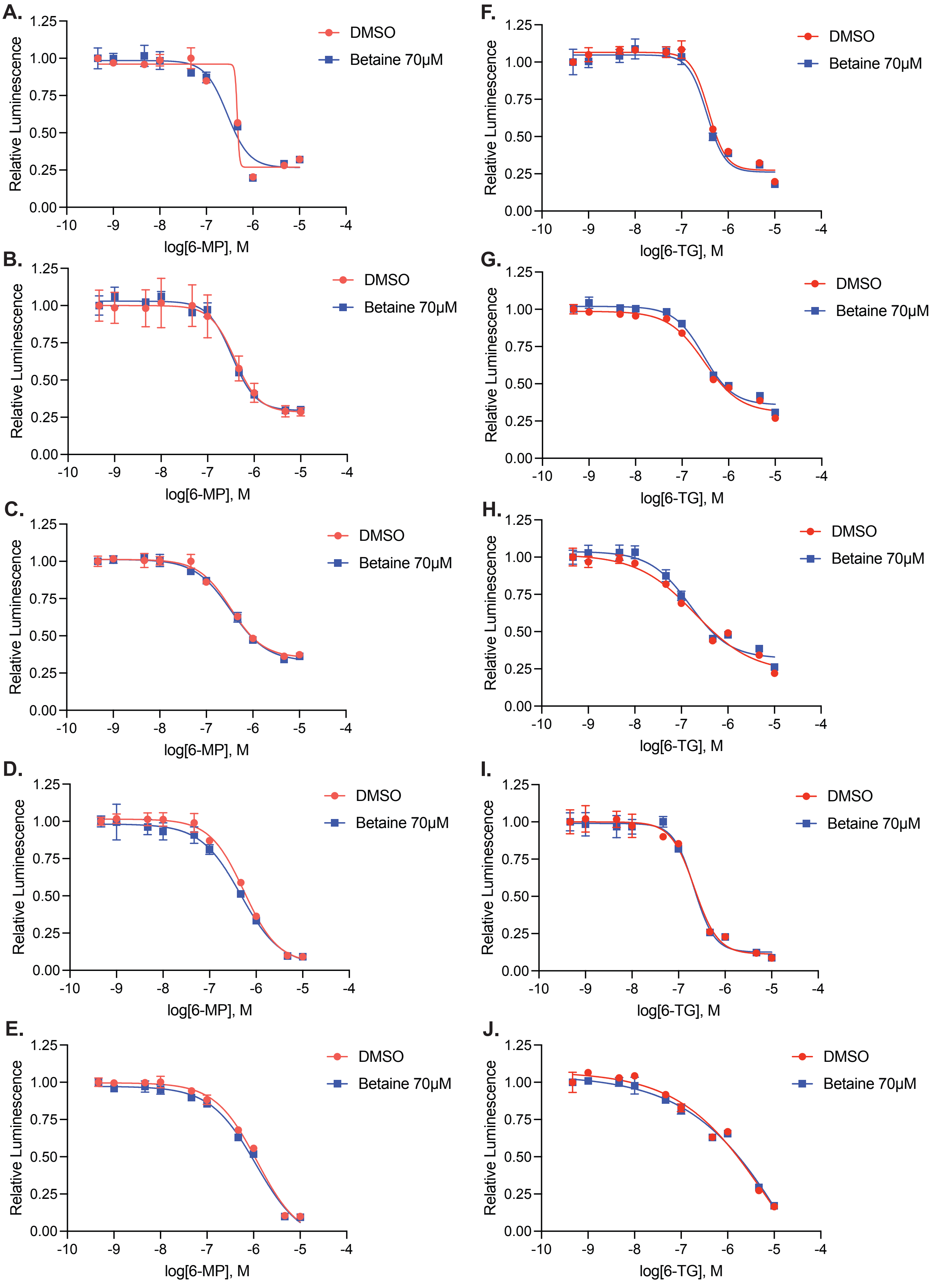

Supplemental Information

Supplemental Figure Legends:

Supplemental Figure 1: Leukemia cell chemotherapy sensitivity in regular compared to human plasma-like media. Doxorubicin (**A-E**) and vincristine (**F-J**) dose-response curves for leukemia cells (NALM-6, **A**, **F**; REH, **B**, **G**; SEM, **C**, **H**; Jurkat, **D**, **I**; CEM, **E**, **J**) in regular or human plasma-like media. Leukemia cell viability was assessed after 48 hours of drug treatment using the CellTiter-Glo Luminescent Cell Viability Assay. Error bars represent the mean ± SD of three technical replicates.

Supplemental Figure 2: Leukemia cell chemotherapy sensitivity in regular compared to human plasma-like media. Cytarabine (**A-E**) and vincristine (**F-J**) dose-response curves for leukemia cells (NALM-6, **A**, **F**; REH, **B**, **G**; SEM, **C**, **H**; Jurkat, **D**, **I**; CEM, **E**, **J**) in regular or human plasma-like media. Leukemia cell viability was assessed after 48 hours of drug treatment using the CellTiter-Glo Luminescent Cell Viability Assay. Error bars represent the mean ± SD of three technical replicates.

Supplemental Figure 3: Leukemia cell chemotherapy sensitivity in regular compared to human plasma-like media. Etoposide (**A-E**) and clofarabine (**F-J**) dose-response curves for leukemia cells (NALM-6, **A**, **F**; REH, **B**, **G**; SEM, **C**, **H**; Jurkat, **D**, **I**; CEM, **E**, **J**) in regular or human plasma-like media. Leukemia cell viability was assessed after 48 hours of drug treatment using the CellTiter-Glo Luminescent Cell Viability Assay. Error bars represent the mean ± SD of three technical replicates.

Supplemental Figure 4: Betaine supplementation of regular media dose not impact leukemia cell sensitivity to thiopurines. 6-MP (**A-E**) and 6-TG (**F-J**) dose-response curves for leukemia cells (NALM-6, **A**, **F**; REH, **B**, **G**; SEM, **C**, **H**; Jurkat, **D**, **I**; CEM, **E**, **J**) in regular media supplemented with betaine 70 μM or DMSO control. Leukemia cell viability was assessed after 48 hours of drug treatment using the CellTiter-Glo Luminescent Cell Viability Assay. Error bars represent the mean ± SD of three technical replicates.
